# Incidental Finding of Heterotaxy Syndrome in a Patient With Pulmonary Embolism: A Case Report and Concise Review

**DOI:** 10.7759/cureus.24326

**Published:** 2022-04-20

**Authors:** Mohamed Mahmoud, Khadija El Kortbi, Hayoung Wang, Joseph Wang

**Affiliations:** 1 Internal Medicine, University of Texas Health Science Center at San Antonio, San Antonio, USA; 2 General Practice, Hassan II University, Faculty of Medicine, Casablanca, MAR; 3 Internal Medicine, University of the Incarnate Word School of Osteopathic Medicine, San Antonio, USA; 4 Internal Medicine/Division of General and Hospital Medicine, University of Texas Health Science Center at San Antonio, San Antonio, USA

**Keywords:** cardiac malformations, congenital hear disease, asplenia with cardiac abnormalities, polysplenia, heterotaxia, pulmonary emboli, situs ambiguus, right atrial isomerism (rai), left atrial isomerism, heterotaxy syndrome (hs)

## Abstract

Heterotaxy syndrome, also called atrial isomerism, is a rare congenital condition in which the internal organs are abnormally arranged across the left-right axis of the body. It is classified into polysplenia syndrome or left atrial isomerism and asplenia syndrome or right atrial isomerism. It is associated with high morbidity and mortality due to the severity of cardiac anomalies. It is important to be aware of the syndrome findings as they can be incidentally found on imaging in adults. Here, we report a case of a 33-year-old female who presented with worsening shortness of breath, found to have a pulmonary embolism, and heterotaxy was incidentally identified on her imaging. A concise review follows.

## Introduction

Heterotaxy syndrome (HS) or atrial isomerism is a spectrum of cardiac and extracardiac abnormalities in which the internal organs are abnormally arranged across the left-right axis of the body [1]. It is divided into polysplenia syndrome or left atrial isomerism (LAI) and asplenia syndrome or right atrial isomerism (RAI). It is typically accompanied by severe cardiac malformations and is associated with high morbidity and mortality [1,2]. The majority die by age five; however, some can reach adulthood with no or mild heart abnormalities. Hence, HS can be identified incidentally on imaging in adults who present for other reasons. Thus, it is important to be aware of this syndrome as it involves multi-organ systems, which could be identified as pathological processes [3].

## Case presentation

**Figure 1:**
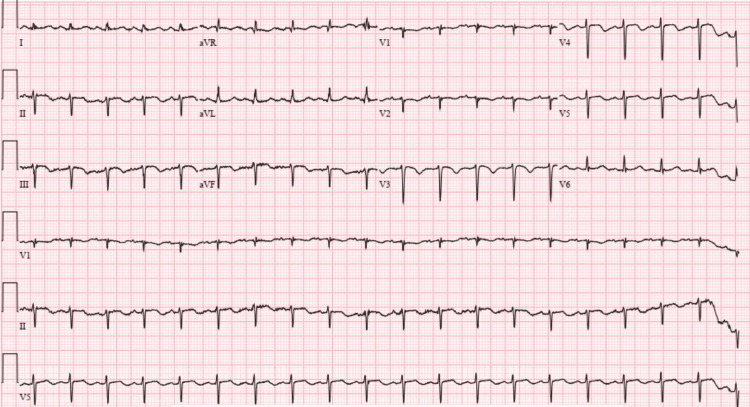
Electrocardiogram (ECG) on admission showing left axis deviation, sinus tachycardia with a heart rate of 117/min, and T-wave inversion in lead II, III, aVF, and V2-V5

**Figure 2:**
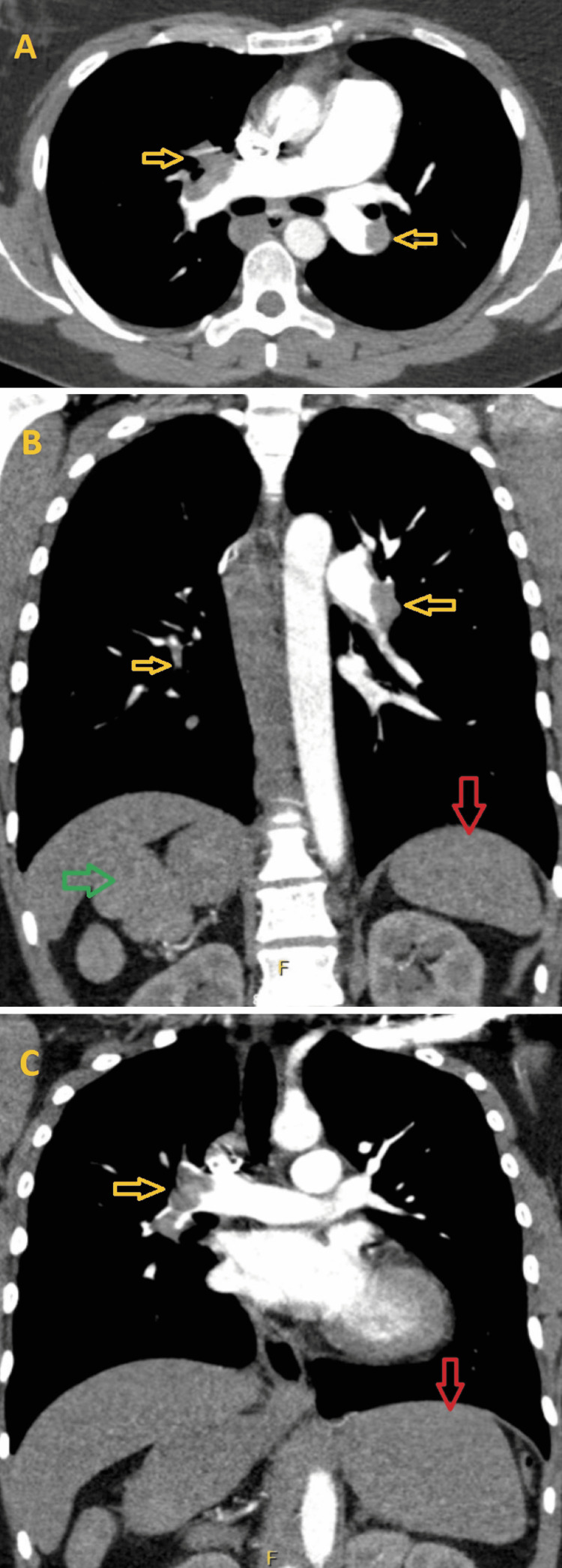
(A) Axial view and (B, C) coronal view of CTPE showing bilateral main pulmonary arterial emboli extending to the lobar and segmental branches (yellow arrows), right-sided polysplenia (green arrow), and a prominent left hepatic lobe (red arrows). CTPE - CT pulmonary embolism study

A 33-year-old female with a history of bronchitis and oral contraceptive (OCP) use presented with progressively worsening shortness of breath over two weeks. She endorsed a productive cough with yellow sputum and denied fever, chills, or chest pain. Vital signs were significant for heart rate 110/min, respiratory rate 22/min, and hypoxia corrected by a 2L nasal canula in the emergency department. Her physical exam was unremarkable except for tachycardia. Labs were pertinent for white blood count 12.5 K/mcL, B-type natriuretic peptide 220 pg/mL, and high sensitivity troponin I 893 ng/L. The respiratory viral panel was negative. An electrocardiogram (ECG) showed sinus tachycardia and T-wave inversion in the inferior leads and leads V2-V5 (Figure 1). A CT (computed tomography) pulmonary angiography showed bilateral main pulmonary arterial emboli extending to the lobar and segmental branches (Figures 2A-2C). Right-sided polysplenia was also noted (Figure 2B).

**Figure 3:**
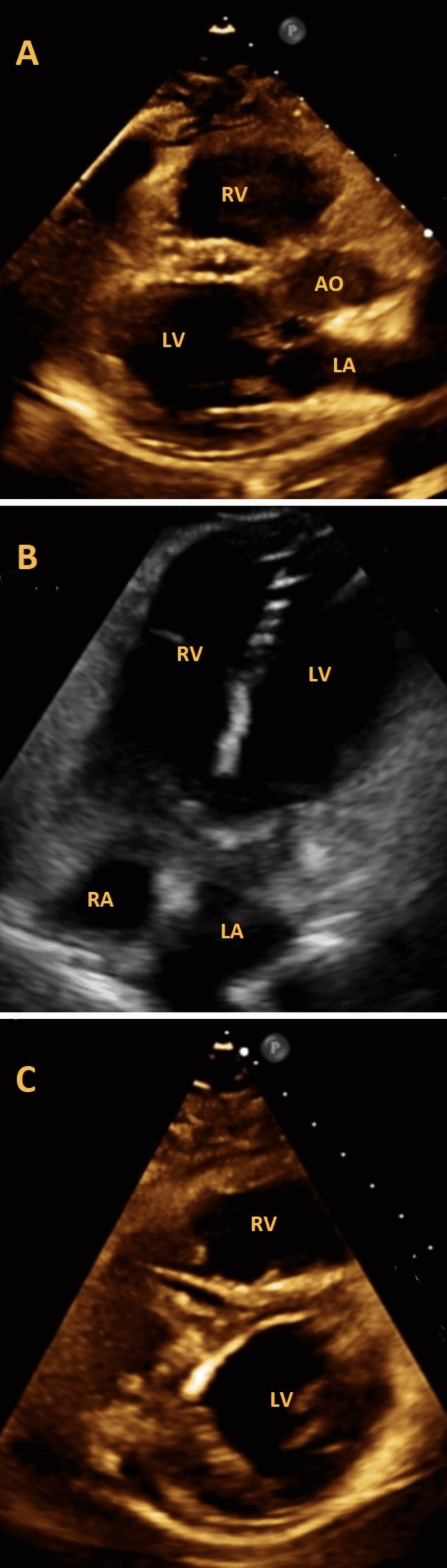
(A) Parasternal long axis, (B) apical four chamber view, and (C) parasternal short axis view showing a dilated right ventricle (RV) on transthoracic echocardiogram (TTE).

An echocardiogram demonstrated right heart strain (Figures 3A-3C), right ventricle hypokinesis, and a negative bubble study. She was started on a heparin drip and underwent catheter thrombolysis with no complications.

**Figure 4:**
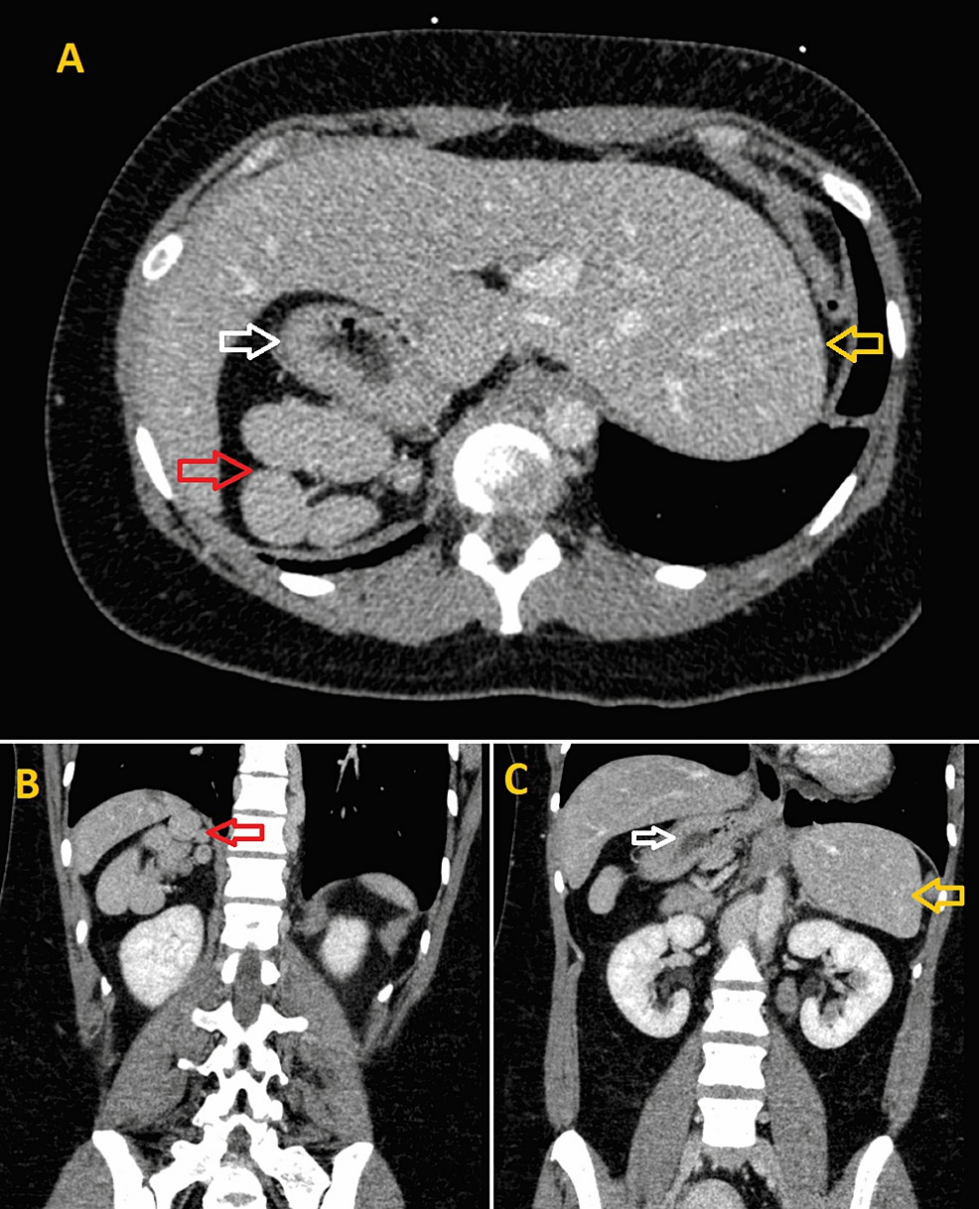
(A) Axial view and (B, C) coronal view of abdominal CT showing a right-sided stomach (white arrows), multiple splenules (polysplenia) without a patent spleen in the right upper quadrant (red arrows), and a prominent left hepatic lobe (yellow arrows), compatible with heterotaxy syndrome.

To further evaluate for intra-abdominal pathology and vascular abnormalities, an abdominal/pelvic CT was obtained and revealed multiple splenules in the right upper quadrant, a right-sided stomach, and a prominent left hepatic lobe, compatible with HS (Figures 4A-4C). Upon further questioning, she denied personal or family history of cardiac abnormalities or HS. The hospital course was uneventful, and she was discharged home on an oral anticoagulant. The patient had an outpatient follow-up with hematology, and the workup for hypercoagulability (factor V, antithrombin III, protein C, protein S, and antiphospholipid antibodies) was negative.

## Discussion

HS, also referred to as atrial isomerism or situs ambiguus, is a rare congenital condition in which the thoracoabdominal organs are abnormally arranged across the left-right axis of the body, and do not conform to situs solitus (normal arrangement of organs) or situs inversus (mirror image of normal arrangement). It typically involves major abnormalities in the cardiovascular, respiratory, and gastrointestinal systems and is associated with significant morbidity and mortality [1]. The estimated incidence is one per 10.000 live birth with a higher prevalence in Asians than Caucasians; however, this number is underestimated as the diagnosis can be missed due to the lack of clinical manifestations [1,2]. Historically, HS has been stratified into two subsets: asplenia or polysplenia syndrome [2]. However, this classification is not useful for describing the associated complex cardiac malformations which represent almost 3% of all congenital heart diseases (CHD) [1].

The segregation of HS into LAI or RAI based solely on atrial appendage morphology does not always indicate other organs' situs. As the atrial appendage morphology is difficult to assess, it is important to assess each organ situs independently. A study of 114 children with HS who underwent non-invasive imaging demonstrated discordance between atrial appendage arrangement, bronchopulmonary branching, and splenic status in >20% of patients [3]. Table 1 summarizes RAI and LAI findings.

**Table 1:** Summary of left atrial isomerism and right atrial isomerism findings AS: Aortic stenosis, ASD: Atrial septal defect, AV: Atrioventricular, AVB: Atrioventricular block, AVNRT: Atrioventricular nodal reentry tachycardia, CoA: Coarctation of the aorta, DORV: Double outlet right ventricle, IVC: Inferior vena cava, LAA: left atrial appendage, LV: Left ventricle, MS: Mitral stenosis, PS: Pulmonary stenosis, RAA: Right atrial appendage, RV: Right ventricle, SA: Sinoatrial, SVC: Superior vena cava, VSD: Ventricular septal defect, VT: Ventricular tachycardia.

	Left Atrial Isomerism	Right Atrial Isomerism
Situs	Bilateral left-sidedness	Bilateral right-sidedness
Cardiac		
Anomalies	More variable	Less variable
Mesocardia	Rare	Common
Atrial appendage	Bilateral LAA morphology	Bilateral RAA morphology (broad-based, triangular shape with a crista terminalis)
Interatrial septum	Variable from intact septum with a fossa ovalis to a common atrium with no septum	Absent and there are large primum and secundum ASD separated by a thin muscular strand
Ventricular morphology	Two good-sized ventricles common	Single RV with a hypoplastic LV common
Ventricular position	Normal D-looping in 80%, L-looping in the rest	Normal D-looping in 65%, L-Looping in the rest
Interventricular septum	Membranous or canal type VSD in 2/3, and an intact septum in 1/3	The inlet septum is hypoplastic with a large VSD
AV valves	Two valves or a common AV valve	Complete AV canal type. Rarely, two separate AV rings.
Sub-pulmonary conus and aortomitral continuity	Present	Present
Left-sided obstruction	AS, MS, and CoA are common	Rare
Pulmonary outflow	PS is rare	Severe PS or atresia is common
Conduction system	Absent or hypoplastic SA node	Duplicated SA node. Two AV nodes may be present.
Associated arrhythmias	AVB, intraventricular conduction delay, sinus node dysfunction, and AVNRT	Atrial flutter, atrial tachycardia, junctional tachycardia, and VT
Vascular		
Coronary sinus	Present	Often absent
SVC	Bilateral	Bilateral
IVC	Often interrupted with azygous or hemiazygos continuation	Interruption of IVC is rare
Great arteries	Often normal. Malposition and DORV are rare	Malposition is common. DORV is rare
Pulmonary veins	Drain directly into the ipsilateral atrium	Usually drain in the SVC and portal system
Respiratory		
Lungs	Two bilobed lungs	Two trilobed lungs
Bronchi	Bilateral hyparterial	Bilateral eparterial
Gastrointestinal and hepatobiliary		
Spleen	Polysplenia	Asplenia or hypoplastic spleen
Liver	Bilobed, often to one side but maybe symmetrical	Symmetrical, Central, and transverse
Biliary tract	Biliary atresia	No abnormalities
Stomach	Right sided	Near midline
Bowel	Malrotation less frequent	Malrotation more frequent

RAI patients present with cyanosis and respiratory distress early in life due to pulmonary outflow tract obstruction and pulmonary venous anomalies (PVA), resulting in the right to left shunting. Asplenia increases the risk of fatal septicemia with encapsulated bacteria [2]. In contrast, LAI patients have a variety of presentations, including asymptomatic due to the wide spectrum of anatomic abnormalities, and they can present in adulthood as they have no or a milder form of heart anomalies. Biliary atresia occurs in 10% and causes jaundice. Both RAI and LAI are associated with gut malrotation leading to intestinal obstruction and bilious vomiting [1,2].

Polysplenia occurs in 2.5 per 100,000 live births and presents in >50% of LAI cases [4]. The majority die by age five due to severe cardiac anomalies, and around 10% reach adulthood with mild or no heart problems. Polysplenia in adults is usually an incidental finding on CT or MRI, such as in our case [5]. Although systemic venous abnormalities such as IVC interruption with azygous continuation are seen in 70% of polysplenia cases [6,7], our patient did not have such abnormalities. Thromboembolism has not been extensively studied in HS; however, one study reported that HS patients with asplenia syndrome and CHD have thrombocytosis and a higher incidence of thromboembolism [8]. However, our case has polysplenia, and using OCPs was likely the predisposing factor for her pulmonary embolism.

HS can occur as a part of other syndromes such as primary cell dyskinesia [6]. Modes of inheritance include autosomal dominant, autosomal recessive, X-linked, or sporadic as in our patient. Mutations in at least 20 genes involved in the left-right laterality have been identified in HS, including ZIC3, Pitx2, NKX2-5, NODAL, CRELD1, LEFTY2, and ACVR2B. Interestingly, ZIC3 mutations have been described in sporadic and X-linked cases [1,7]. The Baltimore-Washington infant study found that maternal diabetes, cocaine use during pregnancy, and family history of malformations are non-genetic risk factors for HS [9].

Prenatal or postnatal diagnosis can be accurately made by echocardiography. In complex cases, three-dimensional echocardiography or fetal MRI is useful in assessing cardiac anomalies. Once the diagnosis is made, further imaging and tests should be done to assess the spleen status. In adults, CT and MRI are excellent in demonstrating non-cardiac abnormalities [1,5].

Cardiac management depends on the severity of cardiac anomalies. Neonates with severe cyanosis would benefit from prostaglandin E1 to maintain the patent ductus arteriosus. Pulmonary congestion and heart failure caused by PVA are managed with pharmacotherapy and supportive care [1].

RAI involves complex cardiac morphology not amenable to biventricular repair, unlike LAI. In that case, staged palliative surgery may be performed [1]. If ventricular dysfunction develops despite surgery, patients will eventually need heart transplantation. Cardiac transplantation techniques have been evolving, and the outcome is now comparable to the pediatric population with situs solitus [10]. LAI patients have an increased risk of complete heart block, sinus node dysfunction, and arrhythmia and may benefit from pacemaker implantation [2].

Cases with asplenia require immunization and should seek medical help early once fever develops. Patients who develop symptoms of intestinal obstruction or biliary atresia should be evaluated promptly for surgical intervention [2].

Many factors impact prognosis and studies on long-term survival are limited [11,12]. A meta-analysis of 36 studies demonstrated that the five-year and 10-year LAI survival were 94% and 83%, respectively, and for RAI 76% and 64%, respectively. In the same analysis, three studies that included HS patients managed in the modern era reported survival of 78% at age three and 70% by age 13 [11]. One study found that tachyarrhythmia in patients with HS is associated with increased mortality compared to bradyarrhythmia [13]. Patients with asplenia, sepsis and biliary atresia also have a worse prognosis [12]. And finally, the biventricular repair is associated with better survival; however, re-intervention is common [14].

## Conclusions

HS involves a wide spectrum of cardiac and non-cardiac abnormalities. In the adult population, HS tends to be an incidental finding. Should there be evidence of HS, further imaging to evaluate for cardiovascular, respiratory, and gastrointestinal abnormalities should be obtained.
